# Metagenomic next-generation sequencing assists in the diagnosis of visceral leishmaniasis in non-endemic areas of China

**DOI:** 10.3389/fcimb.2025.1517046

**Published:** 2025-02-06

**Authors:** Rui Zhao, Guilun He, Lin Xiang, Melinda Ji, Rongheng He, Xudong Wei

**Affiliations:** ^1^ Department of Hematopathy, Henan Institute of Hematology, Cancer Hospital Affiliated to Zhengzhou University, Zhengzhou, Henan, China; ^2^ Science and Technology Service Center, Nanjing Practice Medicine Diagnostics CO., Ltd., Nanjing, Jiangsu, China; ^3^ Department of Translational Research and Cellular Therapeutics, City of Hope, Duarte, CA, United States

**Keywords:** metagenomic next-generation sequencing assists, therapy, leishmaniasis, endemic area, clinical diagnosis

## Abstract

**Introduction:**

Leishmaniasis, a protozoan disease caused by infection by *Leishmania*, is a critical issue in Asia, South America, East Africa, and North Africa. With 12 million cases globally, leishmaniasis is one of the most serious neglected tropical diseases worldwide. Direct identification of infected tissues is currently the primary method of diagnosis; however, the low sensitivity and inconvenience of microscopic examination in detecting amastigotes, parasitic manifestations of *Leishmania*, leads to the possibility of misdiagnosis, delayed diagnosis, and underdiagnosis.

**Methods:**

With the development of metagenomic nextgeneration sequencing (mNGS) technology for pathogen identification, it is possible to detect specific nucleic acid sequences characteristic of *Leishmania* parasites, which opens new avenues for the more accurate diagnosis of leishmaniasis. In this study, we report two cases of leishmaniasis from Henan Province, China, in which *Leishmania* parasites were identified using mNGS technology, massively expediting diagnosis and treatment.

**Results:**

Our report demonstrates that the mNGS method is applicable to peripheral blood samples (PB), which are far more readily available in clinical settings, in addition to bone marrow aspirate samples (BM), which are traditionally used for diagnosis of visceral leishmaniasis.

**Conclusion:**

Our report validates the efficacy of mNGS technology as a rapid and accurate method of diagnosis for leishmaniasis.

## Highlights

- In non-endemic areas, Leishmania can be easily misdiagnosed, resulting in delayed treatment of patients, which can even be life-threatening.
- mNGS has great advantages over traditional methods in the early diagnosis of Leishmania.

## 1 Introduction

Leishmaniasis is a serious protozoan disease that poses a major threat to public health and safety (Hirve et al., 2017).It is characterized by numerous clinical manifestations, which vary based on the host immune reaction and infecting species (Maxfield and Crane, 2024). Zoonotic leishmaniasis most commonly involves phlebotomine sandfly vectors and can be spread from dogs, rodents, hyraxes, and bats to humans (Grevelink and Lerner, 1996; Gebremichael, 2018).

Around 50,000 to 90,000 new cases of visceral leishmaniasis, also known as kala-azar, arise each year, with 90% occurring in rural, impoverished areas throughout Africa and South Asia. However, the global incidence of VL is likely significantly higher, as only an estimated 25% to 45% of total cases are reported (Hirve et al., 2017). If left untreated, over 95% of visceral leishmaniasis (VL) cases are ultimately fatal (Grevelink and Lerner, 1996). Additionally, VL is frequently concurrent with HIV and overlaps significantly in geographical range (Lindoso et al., 2016).

As untimely diagnosis and treatment may result in patient mortality (Alves et al., 2018), ensuring a timely diagnosis and appropriate treatment is crucial for patient health, especially in non-endemic areas (Zhou et al., 2020). In clinical practice, microscopic examination based on the detection of amastigotes, serological tests utilizing enzyme-linked immunosorbent assays (ELISA) to detect anti-*Leishmania* IgG antibodies, and molecular analyses using polymerase chain reaction (PCR) to amplify minute concentrations of *Leishmania*-derived nucleic acids are commonly used methods to diagnose leishmaniasis (Welearegay et al., 2018). However, traditional pathogen detection methods exhibit limited sensitivity and specificity. Serological tests, which are generally more sensitive to visceral leishmaniasis than cutaneous or mucosal leishmaniasis, demonstrate very low sensitivity in diagnosing leishmaniasis-HIV coinfections (Lindoso et al., 2016; De Avelar et al., 2023). Furthermore, while PCR-based nucleic acid amplification is a highly sensitive diagnostic tool for the detection of *Leishmania* (Coulborn et al., 2018). Moreover, detection of multiple species distinctly and concurrently requires multiplex PCR assays, and designing a single PCR cycling protocol to suit each primer pair can present assay constraints (De Brito et al., 2020; De Avelar et al., 2023).

In recent years, metagenomic next-generation sequencing (mNGS) technology has undergone rapid development. In comparison to PCR, mNGS is hypothesis-free and untargeted while exhibiting comparably high sensitivity and a rapid turn-around time (Pallen, 2014). Additionally, mNGS demonstrates significantly higher sensitivity during the early stages of infection than serological assays, circumvents the low accuracy of serological tests when immunodeficiencies are present, and is a considerably more consistent, reliable approach than direct microscopic examination (Chen et al., 2020; Han et al., 2022). Therefore, mNGS offers major advantages over conventional methods in the diagnosis of infectious diseases (Chen et al., 2022; Diao et al., 2022).

In this report, we illustrate the significant role of mNGS in enabling the rapid diagnosis of leishmaniasis through two case reports of misdiagnoses and mistreatment in non-endemic areas. Furthermore, we validate the use of PB, which are considerably easier and far safer to obtain than splenic or BM, for diagnosing leishmaniasis with the mNGS method.

## 2 Materials and methods

Clinical samples for mNGS were obtained from the blood and bronchoalveolar lavage fluid (BALF) of patient 1. BALF was isolated by instilling saline into a pulmonary subsegment, then suctioning and collecting the lavage fluid using a bronchoscope. To isolate the plasma, patient 1’s PB were centrifuged at 2100 g for a duration of 5 minutes. For patient 2, blood and BM were drawn, from which mononucleate cells were isolated and stored prior to DNA extraction.

After collecting and processing the clinical samples, the mNGS process was performed as described subsequently. Sterile deionized water was used in addition to clinical samples as a negative control. After vortexing the samples for 5 minutes with 0.5-mm glass beads (1 g) to lyse cells, complete genomic DNA was extracted from these samples using the TIANamp Micro DNA Kit (DP316, TIANGEN Biotech, Beijing, China) according to the manufacturer’s protocol. Extracted DNA was then sonicated to yield smaller 250-300 bp fragments, as the method followed uses a short-read, shotgun sequencing approach. The concentration and purity of the extracted genomic DNA were assessed using the Qubit and Nanodrop techniques, while the degradation level of genomic DNA was evaluated through 0.8% agarose gel electrophoresis and Agilent 4200 Bioanalyzer (Agilent Technologies Inc.). Qualified single-end DNA libraries were then prepared using the One Shot DNA Library Prep Kit (PDM602, Nanjing Practice Medicine Diagnostics. Co., Ltd., Nanjing, China) and subjected to quality control assessment via an Agilent 2100 biological fragment analyzer. DNA nanoballs (DNBs) were then prepared from amplified single-stranded circular DNA and loaded onto a sequencing chip for high-throughput sequencing using the MGISEQ-200 gene sequencer.

Following sequencing, raw reads were first filtered by removing low-quality reads (PHRED < 20), reads with read-through adapters, reads with multiple uncalled bases (N’s > 2), and short reads (length < 35 bp) using the fastp algorithm (Chen et al., 2018). After adapter trimming and read filtration, low entropy and low-complexity reads consisting mostly of repeats were removed with PRINSEQ software (Schmieder and Edwards, 2011). To account for host-contamination, reads passing quality filters were aligned to the human reference genome (hg38) using Burrows-Wheeler alignment with default parameters inputted into algorithm BWA-MEM (Li and Durbin, 2010). Any single-end reads not mapped to the human genome were then extracted as non-host reads and were used for taxonomic classification. The microbial genome repository used in pathogen identification was synthesized from several public databases: FDA-ARGOS, BV-BRC (version 3.28.9), EuPathDB (Release 52) and NCBI Genbank (Release 242). When constructing the pathogen reference database, only typical representative genomes were retained from the repositories to mitigate redundancy, yielding a final total of 28,516 bacterial, 8,046 viral, 2,076 fungal, and 429 parasitic taxa. Subsequently, the previously extracted non-host reads were aligned to the pathogen reference database, with parameter ‘-Y -h 1000’ inputted into the BWA-MEM algorithm. A mapping quality threshold of 10 was used, to account for the issue of false positives when using BWA [https://www.ncbi.nlm.nih.gov/pmc/articles/PMC2705234/]. The species-specific read number (SSRN) of each species was calculated based on uniquely mapped reads, while multi-mapping reads were assigned to the genus-specific taxon using the lowest common ancestor annotation (LCA) strategy (Huson et al., 2007). The accuracy of classification was further validated using the NCBI BLAST method based on the NR/NT library, in which confirmation was achieved when the BLAST hit with the lowest E-value corresponded to the previously identified target taxon (Johnson et al., 2008).

## 3 Results

Two Chinese patients were admitted to the Cancer hospital of Affiliated to Zhengzhou University, assessed via mNGS analysis, were ultimately diagnosed with visceral leishmaniasis (**Table 1**). mNGS was performed on PB and bronchoalveolar lavage fluid from patient 1, and PB and BM from patient 2. The patients’ medical records are summarized below.

**Table 1:** Summary of patient clinical characteristics.

Patient No./Sex/Age/Ethnicity	Final Diagnosis	Family History	Clinical Symptoms	Travel/Residence in Endemic Area
No. 1/Male/60/Chinese	Visceral leishmaniasis	Normal	Recurrent fevers, pancytopenia, high ferritin, pulmonary infection, atrial fibrillation	None
No.2/Female/10/Chinese	Visceral leishmaniasis	Normal	Recurrent fever, coarse lung texture, splenomegaly	Previous residence in mountainous area with sporadic VL occurrences

### 3.1 Case 1

**Figure 1:**
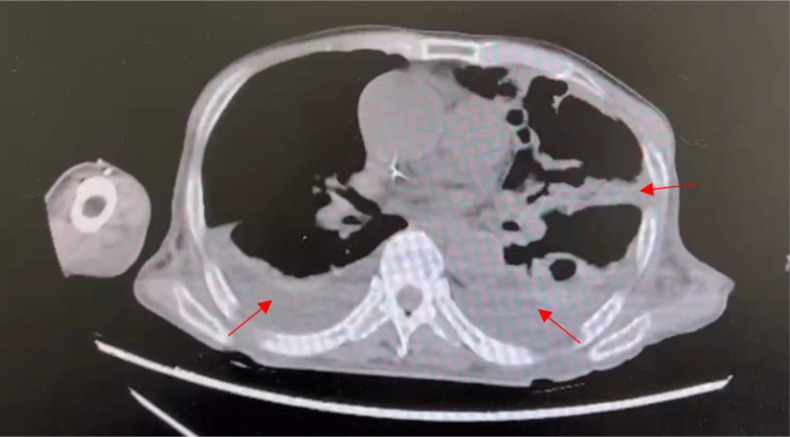
Transverse section from chest CT scan of patient 1 showing pleural effusion, hyperdense regions surrounding the lungs, as indicated by the arrows. Pleural effusion is characteristic of pulmonary infection.

A 60-year-old Chinese male patient with intermittent fevers lasting for more than four months was admitted to the Cancer hospital of Affiliated to Zhengzhou University on December 1st, 2022. Throughout the four-month period, the patient experienced unexplained fevers with a peak temperature of 38.1°C, headaches, lower back pain, acid reflux, and heartburn. No other forms of discomfort, including abdominal pain, diarrhea, black stools, and hematemesis, were reported. On August 21, 2022, the patient went to a local hospital where laboratory test results yielded abnormal values: white blood cell (WBC) count, 1.96×10^9^/L (normal range, [4.0-10]×10^9^/L); red blood cell (RBC) count of 3.31×10^12^/L (normal range, [4.0-5.0]×10^12^/L); hemoglobin (HB) level of 90g/L (normal range, [120-160] g/L); platelet (PLT) count of 37×10^9^/L (normal range, [100-300]×10^9^/L) and ferritin level of 1166 ng/ml (normal range, [40-300] ng/ml). Microscopic examination of the BM showed active hyperplastic bone marrow morphology. The bone marrow biopsy, which revealed a large number of megakaryocytes, suggested hypoplastic anemia. Pleural effusion visible in the chest computed tomography (CT) scan indicated a pulmonary infection (**Figure 1**), which was supported by mNGS analysis revealing *Leishmania* sequences in BALF samples. The patient was diagnosed with hemophagocytic lymphohistiocytosis (HLH), occasional sinus pauses with atrial fibrillation, and a pulmonary infection.

**Figure 2:**
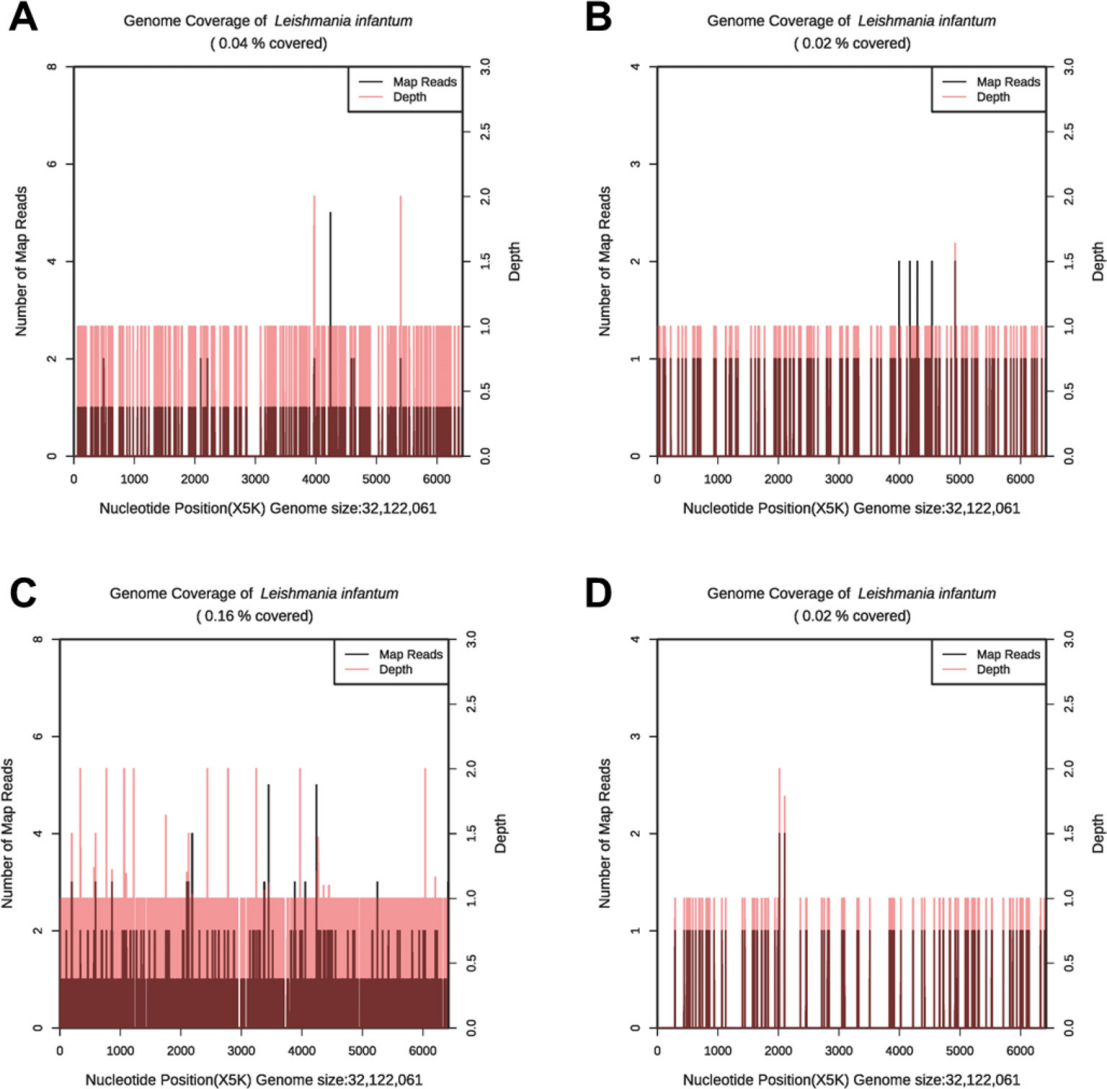
The genome coverage map of *Leishmania infantum* as detected by mNGS in case 1. In the first mNGS test, genome coverage of *Leishmania infantum* in the PB sample was 0.04% **(A)** and 0.02% in BALF **(B)**. In the second mNGS test, the genome coverage of *Leishmania infantum* in the PB sample was 0.16% **(C)** and 0.02% in sputum **(D)**.

On December 4th, 2022, HLH-04 protocol was used to treat the patient, but symptoms did not improve. Pathogen metagenomics test results on the patient’s blood and bronchoalveolar lavage fluid (BALF) indicated infections with *Klebsiella pneumoniae*, *Aspergillus fumigatus*, cytomegalovirus (CMV), and *Leishmania* parasites (**Table 2**, **Figures 2A, B**). However, due to the rarity of the disease in the area and relatively low sequence detection, the patient was not diagnosed with leishmaniasis despite mNGS yielding *Leishmania*-positive results. During hospitalization, the patient was given intravenous imipenem-cilastatin, intravenous capreomycin, intravenous tigecycline, and intravenous ganciclovir to ameliorate infection, but the patient’s condition continued to worsen and platelet counts remained low. In order to quickly identify the cause of the disease, an mNGS test was conducted again two weeks later. Results on the patient's blood and sputum indicated an infection with *Leishmania* parasites once again, and the number of *Leishmania*-derived sequences detected in the blood had increased from 277 in the initial reading to 1045 (**Table 2**, **Figures 2C, D**). As a result, clinical treatment included therapy specifically targeting *Leishmania* parasites in addition to the original plan. After a four-week period in which the patient underwent daily treatment with sodium stibogluconate, administered via intravenous injection at a standard dose of 20 mg/kg, the patient’s symptoms improved. Treatment efficacy was determined based on elimination of fever, pulmonary infection, and return of blood counts to their normal ranges.

**Table 2:** Summary of mNGS results of patient 1.

Sample Type	Date of Testing	Pathogen Detected	Pathogen Category	Relative Abundance	Total Reads	RPM
PB	2022/12/29	*Klebsiella pneumoniae*	B:G-	5.38%	87	2.00
		*Aspergillus flavus*	F	2.17%	1	0.02
		*Human betaherpesvirus 5*	V:DNA	42.86%	3	0.07
		***Leishmania infantum***	Parasites	**16.88%**	**277**	**6.37**
		***Leishmania donovani***	Parasites	**1.58%**	**26**	**0.60**
BALF	2022/12/29	*Klebsiella pneumoniae*	B:G-	6.19%	81	1.83
		*Aspergillus fumigatus*	F	22.37%	17	0.38
		*Aspergillus flavus*	F	14.47%	11	0.25
		*Human betaherpesvirus 5*	V:DNA	100.00%	5	0.11
		***Leishmania infantum***	Parasites	**16.53%**	**143**	**3.23**
		***Leishmania donovani***	Parasites	**0.58%**	**5**	**0.11**
PB	2023/1/11	*Aspergillus fumigatus*	F	16.31%	38	1.26
		*Aspergillus flavus*	F	3.00%	7	0.23
		*Aspergillus niger*	F	1.72%	4	0.13
		*Human betaherpesvirus 5*	V:DNA	94.15%	483	16.04
		*Torque teno virus*	V:DNA	2.85%	15	0.50
		***Leishmania infantum***	Parasites	**16.93%**	**1045**	**34.70**
		***Leishmania donovani***	Parasites	**2.85%**	**176**	**5.84**
Sputum	2023/1/11	*Enterococcus faecium*	B:G+	0.08%	346	11.49
		*Candida lusitaniae*	F	85.69%	497	16.50
		*Aspergillus fumigatus*	F	2.59%	15	0.50
		*Epstein-Barr virus*	V:DNA	48.11%	102	3.39
		*Human betaherpesvirus 7*	V:DNA	32.55%	69	2.29
		*Human betaherpesvirus 5*	V:DNA	16.51%	35	1.16
		***Leishmania infantum***	Parasites	**16.19%**	**102**	**3.39**
		***Leishmania donovani***	Parasites	**0.95%**	**6**	**0.20**

PB: peripheral blood sample, BALF: bronchoalveolar lavage fluid, B:G+: gram-positive bacteria, B:G-: gram-negative bacteria, F: fungal DNA, V:DNA: viral DNA, RPM: reads per million mapped reads. Two changes in the number of sequences of leishmania infection were highlighted in bold.

### 3.2 Case 2

A 10-year-old Chinese female patient with intermittent fevers lasting for more than 2 months was admitted to the Cancer hospital of Affiliated to Zhengzhou University on December 31, 2022. During the two-month period, the patient had experienced frequent high fevers with a maximum temperature of 39.5°C, and a routine blood examination on October 30, 2022 yielded a WBC count of 1.52×10^9^/L (normal range, [4.0-10.0]×10^9^/L), HB level of 75 g/L (normal range, [120-160] g/L), PLT count of 41×10^9^/L (normal range, [100-300]×10^9^/L), and ferritin level of 4180 ng/ml (normal range, [40-300] ng/ml). The concentration of triglycerides in the patient’s bloodstream was determined to be 3.10 mmol/L (normal range, [0.45~1.69 mmol/L]), and an NKA assay measured natural killer cell activity to be 17.54% of lymphocytes (normal range, [4.1-17.3%]). The anti-nuclear antibody spectrum (IHF) test result was positive (+), indicating a potential autoimmune response. Analysis of bone marrow puncture aspirate yielded normal results. Color doppler ultrasound indicated splenomegaly. The patient was ultimately diagnosed with hemophagocytic syndrome (HPS). On November 5, 2022, following the HLH-2018 protocol of Chinese Children’s Histiocytic Group (CCHG-HLH-2018), the patient began treatment with methylprednisolone (10 mg/kg) and ruxolitinib (10 mg q12h po.). Two days later, her body temperature returned to normal. However, on December 24, 2022, the patient’s fever returned at a temperature of 40.0°C. A follow-up routine blood examination supplemented with a C-reactive protein (CRP) test showed a WBC count of 3.27×10^9^/L, HB level of 105 g/L, PLT count of 85×10^9^/L, and CRP level of 133.74 mg/L. A chest CT indicated a coarse lung texture and splenomegaly. The previously prescribed treatment method failed to improve the patient’s fever, suggesting that the patient may have had an infection of another origin.

**Figure 3:**
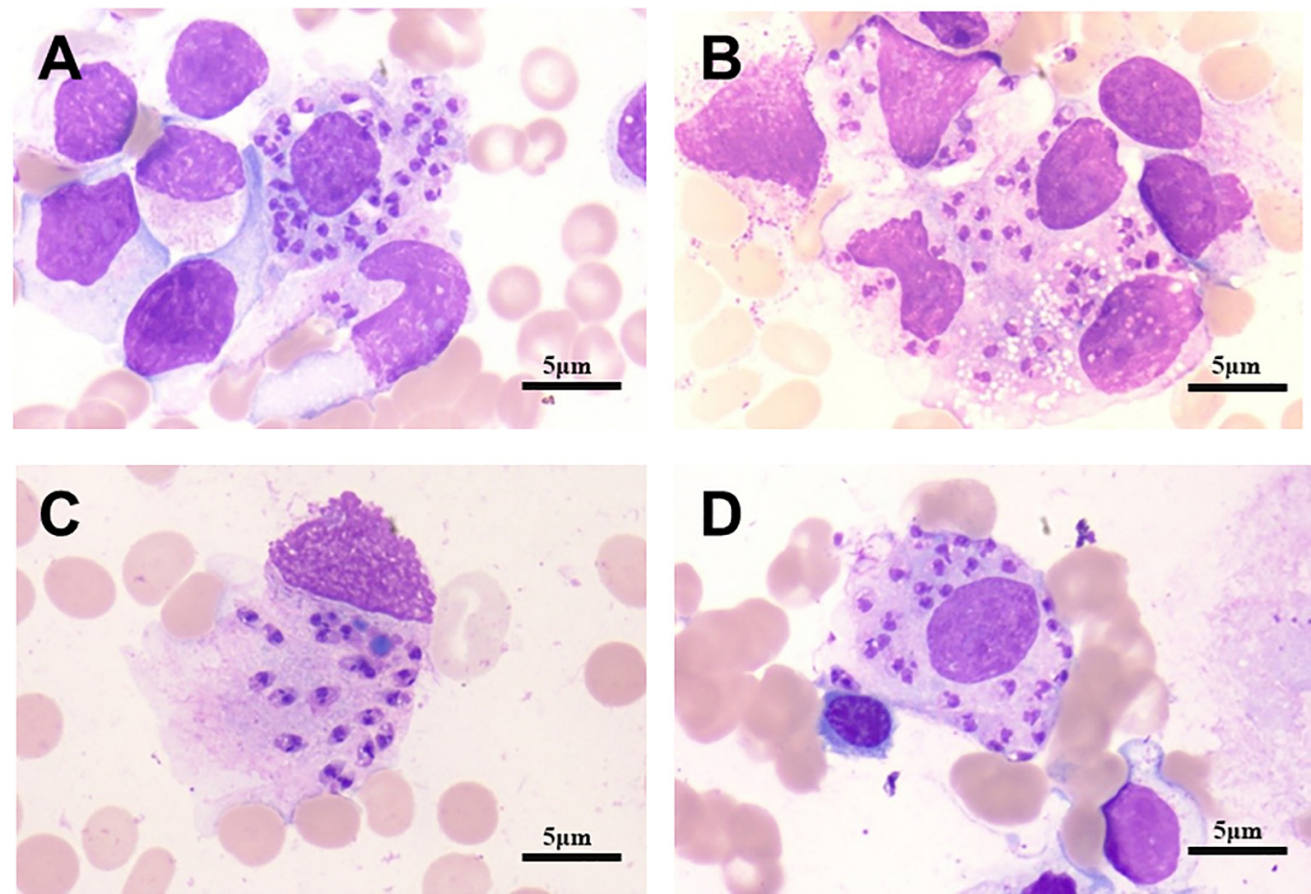
Bone marrow micrographs from patient 2. Active bone marrow hyperplasia, reticular cells, hemophagocytosis and Leishmania amastigotes, as indicated by the arrows, can be seen. **(A)** Macrophages contain numerous Leishman-Donovan bodies. **(B)** Leishman-Donovan body infect more circulating or fixed macrophages. **(C)** The macrophage dies, Leishman-Donovan body are released. **(D)** Macrophages engulf a large number of lydosomes. Cells visualized with H&E stain, 100X magnification. Scale bar represents 5 µm.

**Figure 4:**
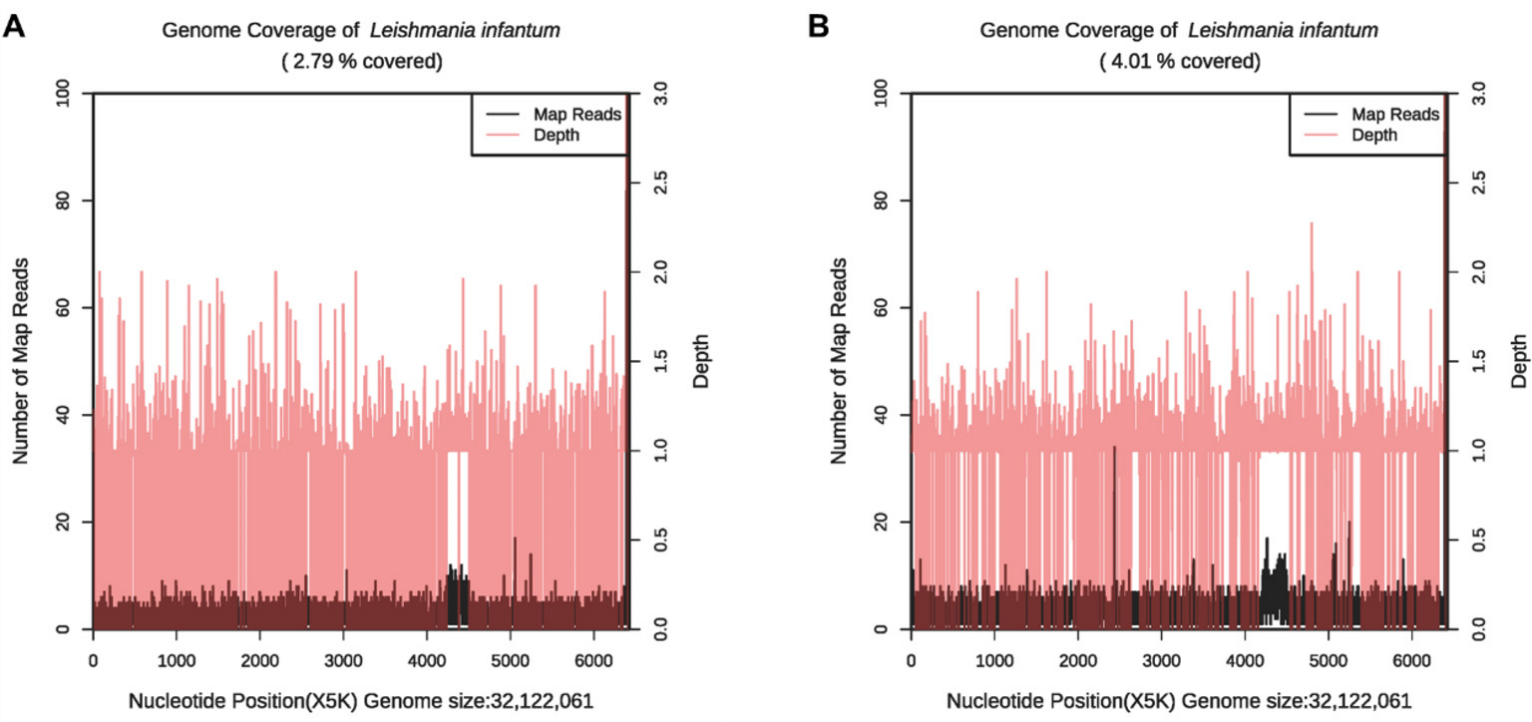
The genome coverage map of *Leishmania infantum* as detected by mNGS in case 2. The genome coverage of *Leishmania infantum* in the PB sample was 2.79% **(A)** and 4.01% in BM **(B)**.

On January 2, 2023, bone marrow and peripheral blood samples were collected and subjected to mNGS. Additionally, a bone marrow sample originating from the same sample used for mNGS was collected and placed under microscopic examination. Result revealed reticular cells, hemophagocytosis and *Leishmania* amastigotes (**Figures 3A–D**), indicating a potential *Leishmania* infection. Consistent with these results, the mNGS results of BM sample showed that *Leishmania*-positive sequences were detected at a high relative abundance of 99.52% (**Table 3**, **Figure 4**). The patient had no abnormal medical or family history, but had previously resided in a mountainous region where sporadic leishmaniasis had previously occurred. The patient was consequently diagnosed with leishmaniasis, and the clinical treatment strategy was adjusted promptly to include daily intravenous injections of sodium stibogluconate (20 mg/kg), leading symptoms to ultimately improve. Two weeks following treatment, microscopic examination of BM revealed no signs of infection. Treatment efficacy was further confirmed based on elimination of fever and return of blood counts to their normal ranges.

**Table 3:** Summary of mNGS results of patient 2.

Sample Type	Date of Testing	Pathogen Detected	Pathogen Category	Relative Abundance	Total Reads	RPM
PB	2023/1/2	*Leishmania infantum*	Parasites	99.49%	15757	650.26
		*Leishmania donovani*	Parasites	0.51%	80	3.30
BM	2023/1/2	*Leishmania infantum*	Parasites	99.52%	20464	844.51
		*Leishmania donovani*	Parasites	0.48%	98	4.04

PB: peripheral blood sample, BM: bone marrow aspirate, RPM: reads per million mapped reads.

## 4 Discussion

Visceral leishmaniasis (VL) was once prevalent in the eastern and central regions of China, with the first reported case of leishmaniasis occurring in 1904. The Henan province, in particular, was once highly endemic for both human and canine visceral leishmaniasis (Yang et al., 2023). However, with the onset of active large-scale prevention and control efforts, the incidence of leishmaniasis in China has decreased annually. According to data published in 2011, the incidence rate of leishmaniasis has decreased to 0.03/100,000 (Guan and Wu, 2014; Lun et al., 2015). Extensive eradication efforts throughout the late 1900s accordingly reduced the incidence of leishmaniasis in Henan. However, beginning in 2016, there has been an upsurge in sporadic cases of local visceral leishmaniasis infections in Henan, with around four cases reported throughout the province each year (Cheng, 2023; Yang et al., 2023).

Based on the species and epidemiological characteristics, visceral leishmaniasis in China is mainly classified into three types: anthroponotic VL (AVL), mountain-type zoonotic VL (MT-ZVL), and desert-type ZVL (DT-ZVL). Research has shown that the average incubation period of visceral leishmaniasis is 3-8 months (Gramiccia et al., 2013; Adamczick et al., 2018). In addition, the clinical symptoms of visceral leishmaniasis such as fever, splenomegaly, and pancytopenia are extensive and overlap with other severe microbial infections, blood diseases, and autoimmune diseases, increasing the difficulty of clinical diagnosis. Furthermore, because of the rarity of the disease in non-endemic areas, underdiagnosis is even more frequent in geographic regions with low incidence (Song et al., 2021).

In this report, both patients developed the disease in the winter without a recent history of travel to endemic areas. Patient 1 had been admitted to another hospital for nearly 3 months, where he had been treated with multiple antibiotics without success. After being transferred to the Cancer hospital of Affiliated to Zhengzhou University, although *Leishmania*-specific sequences were reported in both the peripheral blood and bronchoalveolar lavage fluid in the first mNGS examination, it did not receive significant clinical attention because *Leishmania*-positive reads appeared to be relatively low and *Klebsiella pneumoniae*, *Aspergillus fumigatus*, and cytomegalovirus were concurrently present. It was not until the second mNGS examination of peripheral blood and sputum samples, which revealed an increase in the number of *Leishmania*-positive sequences, that the infection began to be taken seriously. Patient 2 was transferred to the Cancer hospital of Affiliated to Zhengzhou University after ineffective treatment for 2 months at another hospital, where *Leishmania* amastigotes seen in the bone marrow smear suggested leishmaniasis. Both bone marrow and PB from mNGS indicated *Leishmania* infection, and anti-*Leishmania* treatment with sodium stibogluconate was effective.

From the time of initial consultation to the final diagnosis of leishmaniasis, case 1 lasted a total of four and a half months, while case 2 lasted two and a half months. Both cases were initially misdiagnosed as hemophagocytic syndrome and accordingly given chemotherapy to alleviate fever and pancytopenia, causing unnecessary physical, mental, and financial losses to the patients. The considerable delay in diagnosis indicates that traditional diagnostic methods are urgently lacking in reliability, sensitivity, and speed when applied to rare, relapsing infections such as leishmaniasis. Delays are present even in leishmaniasis-endemic areas (Vasconcelos et al., 2024). On average, patients had to visit seven medical services before obtaining an accurate diagnosis (Luz et al., 2019). In non-endemic areas such as the UK, the median time before diagnosis is as long as 6 months (Fletcher et al., 2015). Currently, the clinical diagnosis of visceral leishmaniasis is relatively difficult, with statistical data showing a misdiagnosis rate of around 84.2% (Mondal et al., 2010; Copeland and Aronson, 2015). In case 1 of this report, *Leishmania*-positive reads were initially disregarded despite significantly exceeding the threshold established by Miller et al. for pathogen positivity (10 reads per million) (Miller et al., 2019). Furthermore, for parasites specifically, a stringent-mapped read number (SMRN) of greater than 100 (initial case 1 SMRN of 277) is clinically significant. Because of the rarity of leishmaniasis, especially in a non-endemic area, the possibility of VL was not taken seriously, which contributed to delays in diagnosis and treatment (Diro et al., 2018). Therefore, to ensure an early and accurate diagnosis, mNGS testing should be performed on patients with long-term, re-occurring fevers, abnormal blood counts, and poor responses to previous anti-microbial treatments. If mNGS yields *Leishmania-*positive results, the possibility of leishmaniasis should be considered, and further PCR investigations for different subtypes should be completed.

In recent years, there has been an increase in the use of mNGS to detect *Leishmania* infection (Vogt et al., 2018; Guo et al., 2020; Lin et al., 2021; Gao et al., 2022; Zhang et al., 2022; Chang et al., 2023; Liang et al., 2023).

This report further substantiates the use of mNGS in clinical practice, as it can not only detect *Leishmania* parasites in BM (Chen et al., 2020) but can also be used to test PB, which are significantly more readily obtained. Both patient cases provide incremental evidence of the value of mNGS in detecting specific *Leishmania*-positive sequences from PB, as mentioned previously by Han et al (Han et al., 2022). This further validates the use of PB in identifying leishmaniasis with mNGS, considerably expediting both the sampling and diagnosis process. Interestingly, in the detection results of case 2, we found that the genome coverage map of *Leishmania infantum* (**Figure 4**) may indicate that Chromosome 31 of *Leishmania infantum* in uncultured clinical samples is tetraploid (4 copies), while other chromosomes tend to be diploid (Extended Data **Supplementary Figure S1**). These results demonstrate that the mNGS method can identify the copy number of some chromosomes of pathogens, providing a reference for related research (Domagalska et al., 2019; Reis-Cunha et al., 2024).

Research shows that when screening for pathogens in patients with concurrent immunodeficiency syndromes, bloodstream infections, respiratory infections, or general infections, mNGS has a wide detection range and is suitable for discovering unknown pathogens or co-infections with multiple pathogens (Jiang et al).In particular, HIV-*Leishmania* co-infections are frequent and associated with higher fatality rates, but are often difficult to diagnose. As a result of immunosuppression caused concurrently by HIV and *Leishmania* (Da-Cruz et al., 2006), serology assays relying on host-produced immune factors for diagnosis, such as ELISA assays detecting anti-*Leishmania* IgG, are virtually ineffective. Rapid immunoassays detecting rK39 antigens are also rendered ineffective (Lindoso et al., 2018). mNGS does not rely on the host’s immune status or antibody production. In contrast, delayed-type hypersensitivity (DTH) requires the host to have a certain immune competence, and DTH may produce false-negative results in immunosuppressed or immunodeficient individuals (Gow et al., 2022). A comparison of mNGS and DTH responses after antigen vaccination in the diagnosis of *Leishmania* is shown in **Supplementary Materials Table 1**. mNGS, however, has been previously demonstrated by Tang et al. to be a reliable means of detection, even when leishmaniasis is concurrent with HIV (Tang et al., 2022). In addition, mNGS is not dependent on specific targets, as opposed to targeted molecular methods such as PCR. Most notably, unbiased sampling allows for the unbiased detection of a far broader range of rare, low-level infections and co-infections, rather than quantifying only pre-specified pathogenic sequences. Furthermore, mNGS can theoretically not only identify known pathogens, but also recognize unknown pathogens and potentially even reveal novel pathogens (Han et al., 2019; Zhang et al., 2020). Of course, the sensitivity of PCR in detecting Leishmania in acute VL has been repeatedly demonstrated, so mNGS should be promptly selected as an auxiliary test when multiple previous treatments have failed and the pathogen has not been identified (Chen et al., 2022). Additionally, in a previous case study conducted by Chen et al., peripheral plasma samples yielded zero *Leishmania*-positive reads while BM yielded a 99.6% relative abundance of *Leishmania* (Chen et al., 2020), indicating that blood samples may not always be a reliable predictor of visceral leishmaniasis. The use of blood samples alone in mNGS thus requires further investigation.

Considering the limited number of cases discussed in this report, this study has certain limitations. In the case of patient 1, other diseases were present in addition to leishmaniasis, which could pose potential confounding variables. Because only two cases have been presented, the findings described in this study may not be generalizable to all visceral leishmaniasis cases, as the prognosis of VL may vary depending on factors including comorbidities, geographic region, mode of transmission, primary infecting species, and patient characteristics. Moreover, the follow-up time for patients was not long enough to provide significant post-treatment clinical insights, for instance, regarding the efficacy of treatment strategies in preventing relapse. Regarding case 2, microscopic examination of BM taken two weeks after treatment revealed no signs of infection. Both patients reported no longer experiencing symptoms after a 4-week period and 2-week period for cases 1 and 2, respectively. Patients have been verbally followed up leading up to the present and have reported no recurrence of previous symptoms; however, conclusions cannot be made about the possibility of relapse, as only around 14 months have passed since administration of treatment. Additionally, because the same anti-leishmaniasis treatment (intravenously injected sodium stibogluconate) was administered to both patients, additional research evaluating alternative treatment methods could be beneficial. Further case studies could be valuable in determining the long-term efficacy, potential complications, and patient-specific side effects of alternative therapeutic avenues. Because the relapse rate of leishmaniasis is high, the ability of treatments to ameliorate *Leishmania* infections long-term is an important consideration. Furthermore, personalization of treatment based on genetic markers in the host or the host immune response, both of which can be elucidated by mNGS, can also be explored.

## Data Availability

The original contributions presented in the study are publicly available. This data can be found here: https://www.ncbi.nlm.nih.gov/bioproject/?term=1140367.
