# Supplementary material for: Metagenomic next-generation sequencing assists in the diagnosis of visceral leishmaniasis in non-endemic areas of China
Source: Front Cell Infect Microbiol. 2025 Feb 6;15:1517046. doi: 10.3389/fcimb.2025.1517046 (PMC11839618; doi:10.3389/fcimb.2025.1517046)
Supplement: Supplementary file 1 [file DataSheet1.docx]

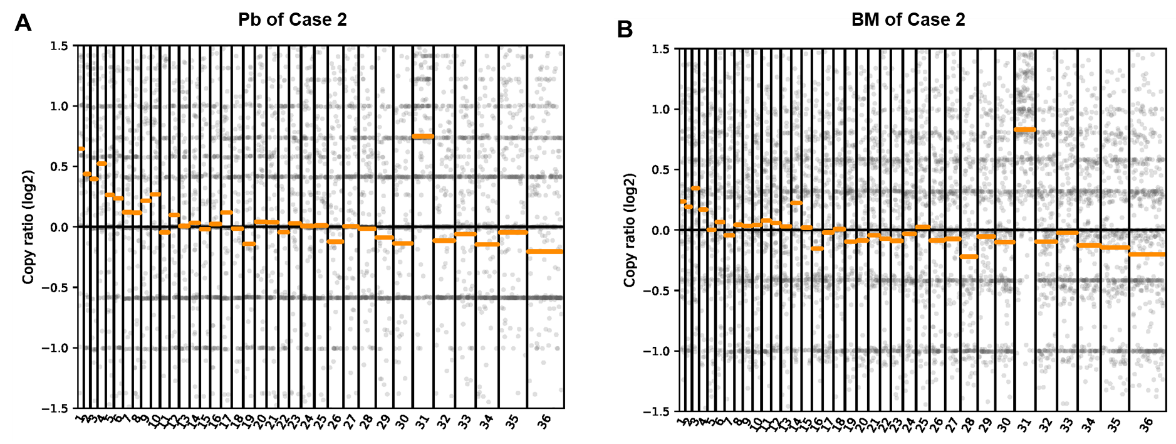


**Supplementary Figure S1** | Quantification of different aneuploidy patterns among *Leishmania infantum* detected by mNGS in case 2. Aneuploidy analysis was carried out by the sliding window size to 5 kbp (gray dots) for *Leishmania infantum* in the PB (A) and BM (B). Copy ratio represents the fold change of expected and observed chromosomal dotation. The median copy ratio is represented in orange.
