# Supplementary material for: Metagenomic next-generation sequencing assists in the diagnosis of visceral leishmaniasis in non-endemic areas of China
Source: Front Cell Infect Microbiol. 2025 Feb 6;15:1517046. doi: 10.3389/fcimb.2025.1517046 (PMC11839618; doi:10.3389/fcimb.2025.1517046)
Supplement: Supplementary file 2 [file Table1.docx]

Supplementary Table 1| Comparison of mNGS and DTH Response Following Antigen Inoculation

| **Feature** | **mNGS** | **DTH Response** |
| --- | --- | --- |
| Sensitivity | High, detects pathogens regardless of immune status. | Host-dependent; lower in immunosuppressed individuals |
| Specificity | High with robust analysis; risk of contamination. | Moderate; risk of cross-reactivity with related pathogens. |
| Host Dependence | Independent of host immunity. | Dependent on the host's immune response. |
| Turnaround Time | 1-3 days (longer for bioinformatics analysis). | 48-72 hours. |
